# Genetic architecture of salt tolerance in a Multi-Parent Advanced Generation Inter-Cross (MAGIC) cowpea population

**DOI:** 10.1186/s12864-022-08332-y

**Published:** 2022-02-05

**Authors:** Waltram Ravelombola, Ainong Shi, Bao-Lam Huynh, Jun Qin, Haizheng Xiong, Aurora Manley, Lingdi Dong, Dotun Olaoye, Gehendra Bhattarai, Bazgha Zia, Huda Alshaya, Ibtisam Alatawi

**Affiliations:** 1 Texas A&M AgriLife Research, 11708 Highway 70 South, 76384 Vernon, TX USA; 2 Department of Soil and Crop Sciences, Texas A&M University, 370 Olsen Blvd., College Station, 77843 TX USA; 3 University of Arkansas, Fayetteville 316 Plant Sciences Building (PTSC), 72701 Fayetteville, AR USA; 4 Department of Nematology, University of California, 92521 Riverside, CA USA; 5 North China Key Laboratory of Biology and Genetic Improvement of Soybean Ministry of Agriculture, Cereal & Oil Crop Institute, National Soybean Improvement Center Shijiazhuang Sub-Center, Hebei Academy of Agricultural and Forestry Sciences, 050031 Shijiazhuang, Hebei China; 6 Cereal & Hebei Academy of Agricultural and Forestry Sciences, No. 162, Hengshanjie Street, 050035 Shijiazhuang, People’s Republic of China

## Abstract

**Background:**

Previous reports have shown that soil salinity is a growing threat to cowpea production, and thus the need for breeding salt-tolerant cowpea cultivars. A total of 234 Multi-Parent Advanced Generation Inter-Cross (MAGIC) lines along with their 8 founders were evaluated for salt tolerance under greenhouse conditions. The objectives of this study were to evaluate salt tolerance in a multi-parent advanced generation inter-cross (MAGIC) cowpea population, to identify single nucleotide polymorphism (SNP) markers associated with salt tolerance, and to assess the accuracy of genomic selection (GS) in predicting salt tolerance, and to explore possible epistatic interactions affecting salt tolerance in cowpea. Phenotyping was validated through the use of salt-tolerant and salt-susceptible controls that were previously reported. Genome-wide association study (GWAS) was conducted using a total of 32,047 filtered SNPs. The epistatic interaction analysis was conducted using the PLINK platform.

**Results:**

Results indicated that: (1) large variation in traits evaluated for salt tolerance was identified among the MAGIC lines, (2) a total of 7, 2, 18, 18, 3, 2, 5, 1, and 23 were associated with number of dead plants, salt injury score, leaf SPAD chlorophyll under salt treatment, relative tolerance index for leaf SPAD chlorophyll, fresh leaf biomass under salt treatment, relative tolerance index for fresh leaf biomass, relative tolerance index for fresh stem biomass, relative tolerance index for the total above-ground fresh biomass, and relative tolerance index for plant height, respectively, with overlapping SNP markers between traits, (3) candidate genes encoding for proteins involved in ion transport such as Na+/Ca2+ K+ independent exchanger and H+/oligopeptide symporter were identified, and (4) epistatic interactions were identified.

**Conclusions:**

These results will have direct applications in breeding programs aiming at improving salt tolerance in cowpea through marker-assisted selection. To the best of our knowledge, this study was one of the earliest reports using a MAGIC population to investigate the genetic architecture of salt tolerance in cowpea.

**Supplementary Information:**

The online version contains supplementary material available at 10.1186/s12864-022-08332-y.

## Background

Cowpea [*Vigna unguiculata* (L.) Walp.] is a diploid legume crop (2*n*=2*x*=22) that is widely grown in various regions such as Africa, Central and South America, Asia, the Middle East, southern Europe, Oceania, and the western and southern United States [1]. The annual worldwide cowpea production is estimated to be 5.4 million tons of cowpea seed with Nigeria being the top producer [2]. Cowpea is grown on a total of 11 million hectares of croplands [3]. Cowpea is a legume that has a multipurpose use. It provides an excellent and affordable source of protein to human [4]. Cowpea seeds contain nutrients that are necessary to human’s heath. One hundred g of cowpea seed contains 6.8 mg of iron, 4.1 mg of zinc, 1.5 mg of manganese, 510 mg of phosphorus, and 1430 mg of potassium [5]. The significant amount of antioxidant compounds within cowpea seeds provides additional nutritional value that would be of interest when incorporated into the diet [6, 7]. In addition to significantly contributing to enhancing the human’s diet, cowpea hay could be used to supplement low quality feed for livestock, a prevalent practice in sub-Sahara Africa [2]. Cowpea also contributes to effective ecosystem management by limiting soil erosion. In fact, with its excellent root architecture plus nitrogen fixation, cowpea can be used as a cover crop that has attracted considerable attention in recent years [8].

Despite of the aforementioned benefits, cowpea production can be substantially limited by abiotic stresses also including soil salinity. Salinity has been reported to increasingly affecting agricultural production worldwide and contributing to an annual loss of 12 billion US dollars [9, 10]. Soil salinity has resulted from the accumulation of cations consisting of K^+^, Mg^2+^, Ca^2+^, and Na^+^ and anions such as NO_3_^−^, HCO_3_^−^, SO_4_^2−^, and Cl^−^ within the soil profile [11]. Soil salinity affects more than 19.6 million ha of croplands in the U.S. and areas facing salinity-related issues have increased [12]. Cowpea cultivation is common in semi-arid areas since cowpea has a better capability to withstand a limited water condition [13]. However, earlier reports suggested that the limited rainfall occurring in semi-arid areas significantly contributed to the salt-related compounds not being effectively leached out from the soil profile, which can exacerbate the effects of salinity on cowpea grown in semi-arid regions [14]. Salinity is also increased by the use of poor quality irrigation water. In the U.S., cowpea cultivation is prevalent in the southern regions [15]. However, irrigation from groundwater in the southern U.S. accounts for more than 66% of the water source used for agricultural activities and can contain up to 1639 mg of Cl^−^ per L of water [16, 17]. A sodium chloride (NaCl) concentration greater than 90 mM, releasing around 526 mg/L of Cl^−^, could significantly reduce cowpea yield [18]. Therefore, salinity could limit cowpea production in relevant southern U.S. areas. Significant cowpea production can also be found in western U.S. in addition to the increasing interest in the use of cowpea as cover crop in this part of the country [8]. The Coachella Valley of California has also been increasingly impacted by salinity, limiting cowpea cultivation expansion in western U.S. [8, 19]. Salinity can also be increased by the overuse of fertilizers or natural factors such as rock weathering [20].

Salinity affects most of development and growth stages of cowpea with germination and seedling stages being the most sensitive stages [21, 22]. Salinity can completely suppress cowpea germination and lead to plant death in cowpea seedlings [22]. In addition, high salt ion concentrations will result in significant reductions in height, biomass, and chlorophyll content, leading to serious physiological impairment within cowpea plants [21]. Breeding for salt-tolerant cowpea cultivars would be one of the most affordable ways to limit the negative effects of salinity on cowpea cultivation. Significant efforts towards investigating salt tolerance in cowpea have been conducted in relatively recent years. Salt tolerance at the germination stage among 151 diverse cowpea accessions have been reported [22]. Another study have identified promising cowpea lines that better withstand salt stress at seedling stage [21]. Molecular markers have substantially assisted plant breeders with rapidly developing cultivars. Our previous article reported the first molecular markers associated with salt tolerance in cowpea [23]. Three SNPs (Scaffold87490_622, Scaffold87490_630, and C35017374_128) were found to be associated with salt tolerance at both seedling and germination stages, and other seven SNPs (Scaffold93827_270, Scaffold68489_600, Scaffold87490_633, Scaffold87490_640, Scaffold82042_3387, C35069468_1916, and Scaffold93942_1089) were reported to be associated with seedling stage-specific salt tolerance in cowpea [23]. The aforementioned research was carried out on an association panel consisting of a diverse cowpea germplasm but having a limited population size, which would reduce the likelihood of finding rare alleles that potentially affect salt tolerance. This can be addressed by conducting a genome-wide association analysis (GWAS) on a multi-parent advanced generation inter-cross (MAGIC) population, as it can increase the frequency of rare alleles while providing significant recombination between the chromosomal Sects. [24–27]. In this study, we used the Bayesian Information and Linkage Disequilibrium Iteratively Nested Keyway (BLINK) model to conduct GWAS. BLINK was built upon the Fixed and Random Model Circulating Probability Unification (FarmCPU) model. In FarmCPU, markers are assumed to be evenly distributed across the genome. However, such an assumption could be easily violated. BLINK relaxed this assumption by incorporating the LD information. The random effect model (REM) part in FarmCPU, which was computationally heavy, was replaced by a second fixed effect model (FEM) in BLINK.

The first MAGIC cowpea population [28] has been developed by using eight founder parents having desirable agronomic traits such as high yield, drought tolerance, and resistance to diseases and insects [28]. However, salt tolerance was not investigated for this MAGIC population while salinity has become an increasing threat to cowpea production worldwide. In addition, genomic selection for salt tolerance remains very limited in cowpea improvement. Investigating the epistatic interactions between markers will further advance our understanding of the genetic mechanism affecting salt tolerance in cowpea. To date, no studies have explored the possibility of epistatic interactions governing salt tolerance in cowpea. The recent advances made in quantitative genetics and modelling permit the identification of epistatic interactions across the plant genome. The study of epistatic interaction between markers can also provide insights to possible gene pathways affecting salt tolerance. These pathways have been demonstrated to affect salt tolerance. For example, in rice, genes involved in membrane sensor proteins, signaling pathways, and electron carriers were found to confer salt tolerance [29]. In cotton, genes involved in the ABA signaling pathway have been reported to enhance salt tolerance [30]. Other reports also highlighted that the salt overly sensitive (SOS) is a key pathway in regulating salt stress in plants [31]. Genes involved in the jasmonate pathway have also been shown to contribute to salt tolerance [32]. The objectives of this study were to evaluate salt tolerance in the cowpea MAGIC population, to identify SNP markers associated with this trait, to assess the accuracy of GS for salt tolerance, and to explore the potential epistatic interactions affecting salt tolerance between SNP markers.

## Results

### Phenotypic data

**Fig. 1:**
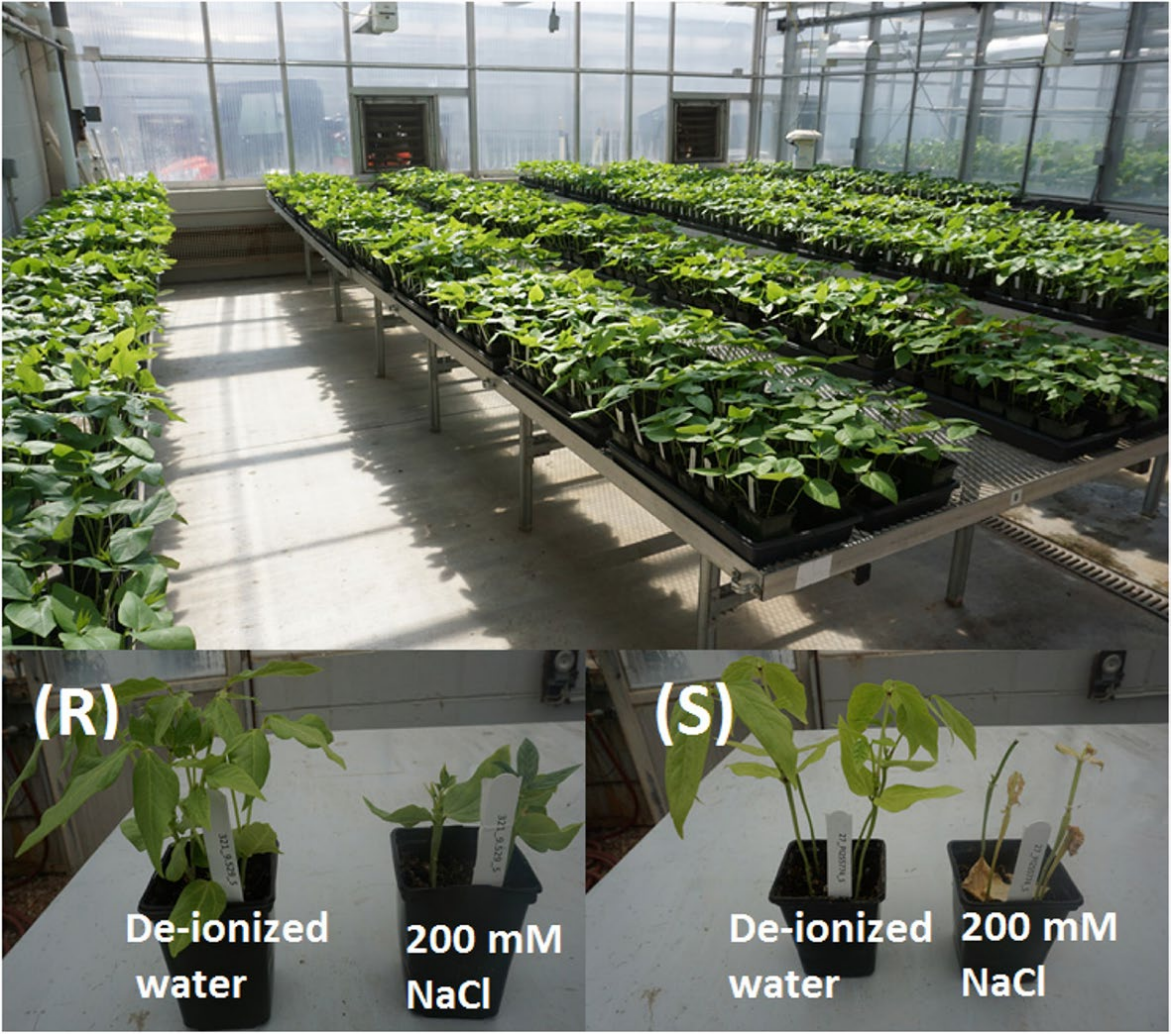
Phenotypic variation in salt-tolerance traits measured in a MAGIC cowpea population. **A** Distribution of the average number of dead plants per pot. **B** Distribution of leaf injury score. **C** Distribution of SPAD chlorophyll of plants under salt stress. **D** Relative tolerance index (RTI) for SPAD chlorophyll. **E** Fresh leaf biomass of plants under salt stress. **F** Relative tolerance index (RTI) for fresh leaf biomass. **G** Relative tolerance index (RTI) for fresh stem biomass. **H** Relative tolerance index (RTI) for total fresh above-ground biomass. **I** Relative tolerance index (RTI) for plant height. The 8 founders were P1: CB27, P2: IT00K-1263, P3: IT82E-18, P4: IT84S-2049, P5: IT84S-2246, P6: IT89KD-288, P7: IT93K-503-1, and P8: Suvita 2

The average number of dead plants per pot varied from 0.0 to 3.0, with an average of 1.0 and a standard deviation of 0.7 (Table S1). The distribution of the average number of dead plants per pot was right-skewed (Fig. 1 A). A significant difference in the average number of dead plants per pot was found among the lines (F-value=15.3, p-value<0.0001) (Table 1), which was expected. Despite the significant line X block interaction effect, the main factor (line) was still analyzed since assessing salt tolerance between lines was the main purpose of the phenotypic evaluation in this study. Of the 242 genotypes evaluated for salt tolerance, 45 did not have any dead plants across 4 replications. In addition, variation in the average number of dead plants per pot was identified as shown in Fig. 1 A. Interestingly, none of the cowpea parents were among the worst 45 lines with plant death. The cowpea parents that were least affected by salt stress in terms of plant death were IT00K-1263 and IT84S-2049 with an average of one dead plant per pot for each. The MAGIC lines with the highest average number of dead plants per pot were MAGIC194 (2.5), MAGIC048 (2.8), IT89KD_288 (3.0), MAGIC074 (3.0), and MAGIC092 (3.0) (Table 2). The broad sense heritability for the average number of dead plants per pot was 74.2%.

**Table 1:** ANOVA for the MAGIC population evaluated under salt tolerance. The evaluated traits were the average number of dead plants per pot (Dead_plants), leaf injury score (Salt_score), SPAD chlorophyll under salt treatment (S_Chloro), relative tolerance index for SPAD chlorophyll (RTI_C), fresh leaf biomass under salt treatment (S_Leaf), relative tolerance index for fresh leaf biomass (RTI_FL), relative tolerance index for fresh stem biomass (RTI_FS), relative tolerance index for total above-ground fresh biomass (RTI_FB), and relative tolerance index for plant height (RTI_H)

Traits	Source	DF	Sum of Squares	Mean Square	Error DF	F Value	Pr > F
Dead_plants	Genotype	241	514.6	2.1	482	15.3	<0.0001
	Block	1	9.3	9.3	482	66.8	<0.0001
	Rep(Block)	2	0.3	0.1	482	0.9	0.397
	Genotype*Block	241	201.4	0.8	482	6	<0.0001
	Residual	482	67.2	0.1	-	-	-
Salt_score	Genotype	241	1166.2	4.8	482	13.2	<0.0001
	Block	1	109.8	109.8	482	299.7	<0.0001
	Rep(Block)	2	0.4	0.2	482	0.6	0.579
	Genotype*Block	241	475.7	2	482	5.4	<0.0001
	Residual	482	176.6	0.4	-	-	-
S_Chloro	Genotype	241	19,857	82.4	482	45.2	<0.0001
	Block	1	327.5	327.5	482	179.7	<0.0001
	Rep(Block)	2	7.8	3.9	482	2.1	0.283
	Genotype*Block	241	6771.9	28.1	482	15.4	<0.0001
	Residual	482	878.4	1.8	-	-	-
RTI_C	Genotype	241	287,943	1194.8	482	26.8	<0.0001
	Block	1	29,566	29,566	482	662.1	<0.0001
	Rep(Block)	2	194.5	97.3	482	2.2	0.312
	Genotype*Block	241	151,075	626.9	482	14	<0.0001
	Residual	482	21,522	44.7	-	-	-
S_Leaf	Genotype	241	472.6	2	482	11.9	<0.0001
	Block	1	159.5	159.5	482	970.1	<0.0001
	Rep(Block)	2	0.9	0.5	482	2.9	0.071
	Genotype*Block	241	254.8	1.1	482	6.4	<0.0001
	Residual	482	79.2	0.2	-	-	-
RTI_FL	Genotype	241	187,356	777.4	482	5.4	<0.0001
	Block	1	92,398	92,398	482	638.6	<0.0001
	Rep(Block)	2	654.8	327.4	482	2.3	0.1052
	Genotype*Block	241	90,725	376.5	482	2.6	<0.0001
	Residual	482	69,744	144.7	-	-	-
RTI_FS	Genotype	241	154,541	641.2	482	4.3	<0.0001
	Block	1	62,106	62,106	482	419	<0.0001
	Rep(Block)	2	481.6	240.8	482	1.6	0.1981
	Genotype*Block	241	80,826	335.4	482	2.3	<0.0001
	Residual	482	71,449	148.2	-	-	-
RTI_FB	Genotype	241	134,866	559.6	482	6.5	<0.0001
	Block	1	84,129	84,129	482	980.8	<0.0001
	Rep(Block)	2	30.1	15	482	0.2	0.6109
	Genotype*Block	241	70,323	291.8	482	3.4	<0.0001
	Residual	482	41,344	85.8	-	-	-
RTI_H	Genotype	241	34,176	141.8	482	6.9	<0.0001
	Block	1	8263.1	8263.1	482	401	<0.0001
	Rep(Block)	2	114.7	57.3	482	2.8	0.0629
	Genotype*Block	241	15,626	64.8	482	3.1	<0.0001
	Residual	482	9931.3	20.6	-	-	-

**Table 2:** List of top 5 genotypes and 5 least performers for average number of dead plants per plot (DeadPlants), the leaf injury score under salt treatment (Score), SPAD chlorophyll under salt treatment (StressSPADChloro), relative tolerance index for SPAD chlorophyll (RTI_C), fresh leaf biomass under salt treatment (StressLeaf), relative tolerance index for fresh leaf biomass (RTI_FL), relative tolerance index for fresh stem biomass (RTI_FS), relative tolerance index for total above-ground fresh biomass (RTI_FB), and relative tolerance index for plant height (RTI_H). Sd represents the standard deviation across 4 replications

**Plant_ID**	**DeadPlants**	**Sd**	**Plant_ID**	**Score**	**Sd**	**Plant_ID**	**StressSPADChloro**	**Sd**
MAGIC001	0	0	MAGIC208	0.5	0.6	MAGIC092	7.1	5.1
MAGIC008	0	0	MAGIC027	1.3	1	MAGIC048	8.5	5.3
MAGIC009	0	0	MAGIC040	1.3	0.5	MAGIC194	10.2	6.9
MAGIC012	0	0	MAGIC062	1.3	0.5	MAGIC110	11.4	5.1
MAGIC027	0	0	MAGIC236	1.3	0.5	MAGIC122	12	5.6
MAGIC194	2.5	0.6	MAGIC259	6	0.8	MAGIC236	44	8.6
MAGIC048	2.8	0.5	MAGIC298	6	0.8	MAGIC311	45.3	2.8
IT89KD_288	3	0	MAGIC194	6	0	MAGIC027	46	4.6
MAGIC074	3	0	MAGIC048	6.3	0.5	MAGIC008	47.2	1.9
MAGIC092	3	0	MAGIC092	6.5	0.6	MAGIC208	51.5	6
**Plant_ID**	**RTI_C**	**Sd**	**Plant_ID**	**StressLeaf**	**Sd**	**Plant_ID**	**RTI_FL**	**Sd**
MAGIC092	23.6	16.9	IT84S_2246	0.5	0.1	MAGIC073	10	8
MAGIC048	27.4	16.1	MAGIC092	0.5	0.4	MAGIC130	17.4	10
MAGIC110	28.9	11.3	MAGIC048	0.6	0.4	IT84S_2246	17.9	4.8
MAGIC074	30.8	27.7	MAGIC073	0.6	0.4	MAGIC110	21.2	12
MAGIC194	32.2	20.5	Suvita_2	0.7	0.1	MAGIC207	26.2	14.1
MAGIC236	104	10.7	MAGIC027	3.8	0.6	MAGIC201	88	4.4
MAGIC008	104.5	8.2	MAGIC187	3.8	0.5	MAGIC265	90.6	4.1
MAGIC343	105.8	9.2	MAGIC271	3.8	1	MAGIC188	92.5	6.5
MAGIC311	107.8	10.9	MAGIC336	3.8	1.8	MAGIC264	93.1	5.4
MAGIC119	108.1	10	MAGIC208	4.2	0.7	MAGIC177	93.2	3.5
**Plant_ID**	**RTI_FS**	**Sd**	**Plant_ID**	**RTI_FB**	**Sd**	**Plant_ID**	**RTI_H**	**Sd**
MAGIC207	23	12.9	MAGIC130	9.6	12.2	MAGIC074	54.6	2.2
MAGIC130	24.6	11.4	MAGIC148	12.4	13.8	MAGIC153	56.7	2.4
MAGIC089	27.5	18.4	MAGIC134	12.6	13.7	MAGIC206	57.5	11.1
MAGIC119	27.7	5	MAGIC259	13.2	15.4	MAGIC072	58.4	1.4
MAGIC073	28	25.1	MAGIC146	13.8	15.6	MAGIC030	58.8	5.1
MAGIC238	86.6	7	MAGIC199	46.5	53.1	MAGIC077	85.8	10.6
MAGIC271	87.2	5.5	MAGIC242	46.6	53.6	MAGIC138	86.5	10.6
MAGIC343	88.5	5.5	MAGIC282	46.8	53.6	MAGIC280	86.9	9.1
MAGIC270	88.7	9.1	MAGIC187	47	53.6	MAGIC117	87.3	5.8
MAGIC181	89.9	4.1	MAGIC188	47.9	54.4	MAGIC199	89.5	6.3

Leaf injury score was approximately normally distributed (Fig. 1B) and ranged between 0.5 and 6.5, with an average of 3.6 and a standard deviation of 1.1 (Table S1). The lines differed significantly in leaf injury (F-value=13.2, p-value<0.0001) (Table 1). Lines with the lowest leaf injury score were MAGIC208 (0.5), MAGIC027 (1.3), MAGIC040 (1.3), MAGIC062 (1.3), and MAGIC236 (1.3) (Table 2),.Lines with the highest leaf injury score were MAGIC259 (6.0), MAGIC298 (6.0), MAGIC194 (6.0), MAGIC048 (6.3), MAGIC092 (6.5) (Table 2), which were the most susceptible genotypes in terms of leaf injury score. None of the MAGIC parents were among the most tolerant and the most susceptible groups. The parent that was the most tolerant to salt stress in terms of leaf injury score were IT00K-1263 (3.3), whereas the one that was the most susceptible was IT89KD-288 (5.8) (Fig. 1B). The broad sense heritability (*H*) for leaf injury score under salt stress was 72.6%.

The distribution of leaf SPAD chlorophyll under salt treatment was approximately normally distributed as shown in Fig. 1 C. Leaf SPAD chlorophyll was significantly different among the MAGIC lines (F-value=45.2, p-value<0.0001) (Table 1). The lines with the highest leaf SPAD chlorophyll under salt stress were MAGIC208 (51.5), MAGIC008 (47.2), MAGIC027 (46.0), MAGIC311 (45.3), and MAGIC236 (44.0), whereas those with the lowest leaf SPAD chlorophyll under salt stress were MAGIC122 (12.0), MAGIC110 (11.4), MAGIC194 (10.2), MAGIC048 (8.5), and MAGIC092 (7.1) (Table 2). The MAGIC parent with the highest leaf SPAD chlorophyll under salt treatment was IT84S-2246 (13.6), which was the most susceptible parent in terms of leaf SPAD chlorophyll under salt treatment. The MAGIC parent with the highest leaf SPAD chlorophyll under salt stress was IT93K-503-1 (21.9) (Table 2). The broad sense heritability (*H*) for leaf SPAD chlorophyll under salt treatment was 78.9%.

Relative tolerance index for leaf SPAD chlorophyll (RTI_C) was approximately normally distributed (Fig. 1D). RTI_C was significantly different among genotypes (F-value=26.8, p-value<0.0001) (Table 1). The top 5 lines with the highest RTI_C were MAGIC119 (108.1%), MAGIC311 (107.8%), MAGIC343 (105.8%), MAGIC008 (104.5%), and MAGIC236 (104.0%) (Table 2). Their RTI_C was greater than 100%, indicating that they were highly salt-tolerant based on RTI_C and the leaf SPAD chlorophyll content under salt stress was greater than that of under non-salt stress. The lines with the lowest RTI_C were MAGIC194 (32.2%), MAGIC074 (30.8%), MAGIC110 (28.9%), MAGIC048 (27.4%), and MAGIC092 (23.7%) (Table 2), suggesting that these genotypes were the most susceptible to salt stress based on RTI_C in this population. The MAGIC parent with the highest RTI_C was IT84S-2049 (68.8%), whereas the one with the lowest RTI_C was IT84S-2246 (42.7%). The broad sense heritability (*H*) for RTI_C was 63.6%.

Fresh leaf biomass under salt stress is also a good character for assessing salt tolerance in cowpea at the seedling stage. In this study, fresh leaf biomass of cowpea plants under salt treatment was approximately normally distributed (Fig. 1E). Fresh leaf biomass ranged between 0.5 and 4.2 g, with an average of 2.1 g and a standard deviation of 0.7 g (Table S2). Under salt stress, a significant difference in fresh leaf biomass was identified among the lines (F-value=11.9, p-value<0.0001) (Table 1). Lines with the highest fresh leaf biomass under salt stress were MAGIC208 (4.2 g), MAGIC336 (3.8 g), MAGIC271 (3.8 g), MAGIC187 (3.8 g), and MAGIC027 (3.8 g), whereas those with the lowest fresh leaf biomass under salt stress were Suvita 2 (0.7 g), MAGIC073 (0.6 g), MAGIC048 (0.6 g), MAGIC092 (0.5 g), and IT84S_2246 (0.5 g) (Table 2). The broad sense (*H*) heritability for fresh leaf biomass of cowpea plants grown under salt treatment was 61.3%.

The relative tolerance index for fresh leaf biomass (RTI_FL) varied from 10.0 to 93.2%, with an average of 56.8% and a standard deviation of 13.9% (Table S2). RTI_FL was normally distributed as shown in Fig. 1 F. ANOVA indicated a significant difference among lines in RTI_FL (F-value=5.4, p-value<0.0001) (Table 1). Lines with the highest RTI_FL were MAGIC177 (93.2%), MAGIC264 (93.1%), MAGIC188 (92.5%), MAGIC265 (90.6%), and MAGIC201 (88.0%), which were the most tolerance in terms of relative tolerance index for fresh leaf biomass (Table 2). Lines that were most susceptible to salt stress in terms of RTI_FL were MAGIC207 (26.2%), MAGIC110 (21.2%), IT84S_2246 (17.9%), MAGIC130 (17.4%), and MAGIC073 (10.0%) (Table 2). The MAGIC parent with the highest RTI_FL was Suvita 2 (59.0%). RTI_FL values for the MAGIC parents were scattered across the distribution of RTI_FL for this population (Fig. 1 F). The broad sense heritability (*H*) for RTI-FL was 64.1%.

Relative tolerance index for fresh stem biomass (RTI_FS) was normally distributed (Fig. 1G). RTI_FS varied from 23.0 to 89.9%, with an average of 54.7% and a standard deviation of 12.7% (Table S2). A significant difference in terms of RTI_FS was found among the cowpea lines investigated for salt tolerance in this study (F-value=4.3, p-value<0.0001) (Table 1). The top performing MAGIC lines in terms of RTI_FS were MAGIC181 (89.9%), MAGIC270 (88.7%), MAGIC343 (88.5%), MAGIC271 (87.2%), and MAGIC238 (86.6%), and the MAGIC lines that were the least performing in terms of RTI_FS were MAGIC073 (28.0%), MAGIC119 (27.7%), MAGIC089 (27.5%), MAGIC130 (24.6%), and MAGIC207 (23.0%) (Table 2). The MAGIC parent with the highest RTI_FS was IT89KD-288 (77.5%), whereas the one with the lowest RTI_FS was IT82E-18 (40.6%) (Table S2). The broad sense heritability (*H*) for RTI_FS was 59.9%.

Relative tolerance index for total above-drought fresh biomass (RTI_FB) was normally distributed as shown in Fig. 1 H. RTI_FB ranged between 9.6% and 47.9%, with an average of 35.5% and a standard deviation of 7.6% (Table S3). The MAGIC lines that were the most tolerant to salt stress in terms of RTI_FB were MAGIC188 (47.9%), MAGIC187 (47.0%), MAGIC282 (46.8%), MAGIC242 (46.6%), and MAGIC199 (46.5%), whereas those that were the most susceptible to salt stress based on RTI_FB were MAGIC146 (13.8%), MAGIC259 (13.2%), MAGIC134 (12.6%), MAGIC148 (12.4%), and MAGIC130 (9.6%) (Table 2). The parent with the highest RTI_FB was Suvita 2 (41.3%), whereas the one with the lowest RTI_FB was IT82E-18 (14.0%). The broad sense heritability (*H*) for RTI_FB was 61.5%.

The distribution of relative tolerance index for plant height (RTI_H) was approximately normal (Fig. 1I). RTI_H varied from 54.6 to 89.5%, with an average of 73.1% and a standard deviation of 6.0% (Table S3). A significant difference in RTI_H was identified among the lines (F-value=6.9, p-value<0.0001) (Table 1). The MAGIC lines with the highest RTI_H were MAGIC199 (89.5%), MAGIC117 (87.3%), MAGIC280 (86.9%), MAGIC138 (86.5%), and MAGIC077 (85.8%) (Table 2), thus the most tolerant to salt stress based on RTI_H. Lines most susceptible to salt stress in terms of RTI_H were MAGIC030 (58.8%), MAGIC072 (58.4%), MAGIC206 (57.5%), MAGIC153 (56.7%), and MAGIC074 (54.6%) (Table 2). The parent with the highest RTI_H was IT93K-503-1 (79.8%), whereas the one with the lowest RTI_H was IT84S-2246 (59.2%). The broad sense heritability (*H*) for RTI_H was 67.2%.

## Correlation analysis

The average number of dead plants per pot was strongly correlated with the salt injury score (*r*=0.9, P < 0.001). In addition, a strong and negative correlation was found between the average number of dead plants per pot and the leaf SPAD chlorophyll under salt stress (*r*=-0.8, P < 0.001), and between the average number of dead plants per pot and the relative tolerance index for leaf SPAD chlorophyll (*r*=-0.8, P < 0.001) (Table 3). A high and negative correlation was also identified between the average number of dead plants per pot and fresh leaf biomass under salt stress (*r*=-0.6, P < 0.001). The average number of dead plants per pot was significantly correlated with the relative tolerance index for fresh stem biomass (*r*=-0.20, P < 0.001), relative tolerance index for total above-ground fresh biomass (*r*=-0.30, 0.001), and relative tolerance index for plant height (*r*=-0.10, non-significant) (Table 3). The relative tolerance index for leaf SPAD chlorophyll was moderately correlated with fresh leaf biomass under salt stress (*r*=0.50, P < 0.001), but the relative tolerance index for leaf SPAD chlorophyll was not correlated with the relative tolerance index for plant height (*r*=-0.10, non-significant), indicating that the mechanism for tolerance to plant height reduction and leaf SPAD chlorophyll reduction under salt stress could be different. The trait having the highest correlation with relative tolerance index for plant height was fresh stem biomass (*r*=0.40, P < 0.001) (Table 3).

**Table 3:** Persons’ correlation coefficients for the traits evaluated for salt tolerance in a MAGIC population. Traits consisted of average number of dead plants per plot (DeadPlants), the leaf injury score under salt treatment (Score), SPAD chlorophyll under salt treatment (StressSPADChloro), relative tolerance index for SPAD chlorophyll (RTI_C), fresh leaf biomass under salt treatment (StressLeaf), relative tolerance index for fresh leaf biomass (RTI_FL), relative tolerance index for fresh stem biomass (RTI_FS), relative tolerance index for total above-ground fresh biomass (RTI_FB), and relative tolerance index (RTI) for plant height (RTI_Height)

	DeadPlants	Score	StressSPADChloro	RTI_C	StressLeaf	RTI_FL	RTI_FS	RTI_FB	RTI_H
**DeadPlants**	1	-	-	-	-	-	-	-	-
**Score**	0.9***	1	-	-	-	-	-	-	-
**StressSPADChloro**	-0.8***	-0.9***	1	-	-	-	-	-	-
**RTI_C**	-0.8***	-0.8***	0.9***	1	-	-	-	-	-
**StressLeaf**	-0.6***	-0.6***	0.6***	0.5***	1	-	-	-	-
**RTI_FL**	-0.4***	-0.4***	0.4***	0.4***	0.7***	1	-	-	-
**RTI_FS**	-0.2***	-0.2**	0.3***	0.3***	0.5***	0.6***	1	-	-
**RTI_FB**	-0.3***	-0.2***	0.3***	0.3***	0.5***	0.6***	0.6***	1	-
**RTI_H**	-0.1	-0.1	0.2*	0.1	0.2**	0.3***	0.4***	0.3***	1

*, **, *** indicates a significant correlation at *P* < 0.05, 0.01, and 0.001, respectively

**Fig. 2:**
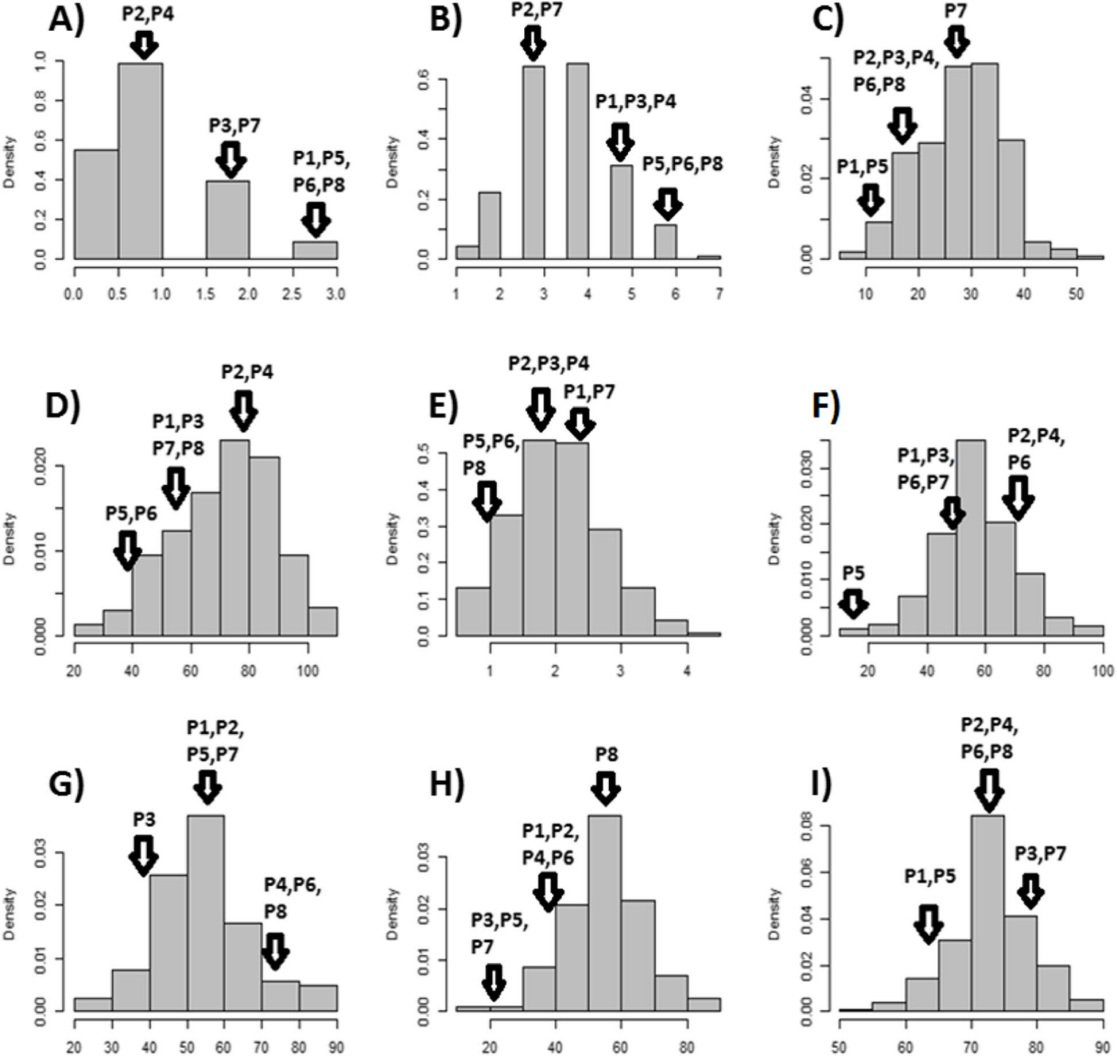
Chord diagram showing the pairwise correlation between traits evaluated under salt tolerance in a MAGIC cowpea population. The legends outside the chord diagram correspond to the different traits (RTI_biomass= relative tolerance index for total above-ground fresh biomass, RTI_Height= relative tolerance index for plant height, Dead= average number of dead plants per pot, Score= leaf injury score, StressSPADChloro= leaf SPAD chlorophyll under salt stress, RTI_SPADChloro= relative tolerance index for leaf SPAD chlorophyll, StressLeaf= fresh leaf biomass under salt stress, RTI_Leaf= relative tolerance index for fresh leaf biomass, and RTI_Stem= relative tolerance index for fresh stem biomass). The width of the link between traits was proportional to the absolute value of the Pearson’s correlation coefficient. The score on the outer circle corresponds to the thickness of each chord

The pairwise relationship that was based on the Pearson’s correlation coefficient for the traits evaluated under salt stress was visualized used a chord diagram (Fig. 2). The thicker the link between traits the higher the Person’s correlation coefficient. Traits with the thickest link end were the average number of dead plants per pot, leaf injury score, leaf SPAD chlorophyll under salt stress, and relative tolerance index for leaf SPAD chlorophyll (Fig. 2), suggesting the possibility of common pathway(s) for salt tolerance mechanism for these traits. Traits with the thinnest link end were relative tolerance index for fresh stem and plant height, indicating that the mechanism for salt tolerance could be independent from the other traits evaluated in this study.

## GWAS and candidate gene identification

**Fig. 3:**
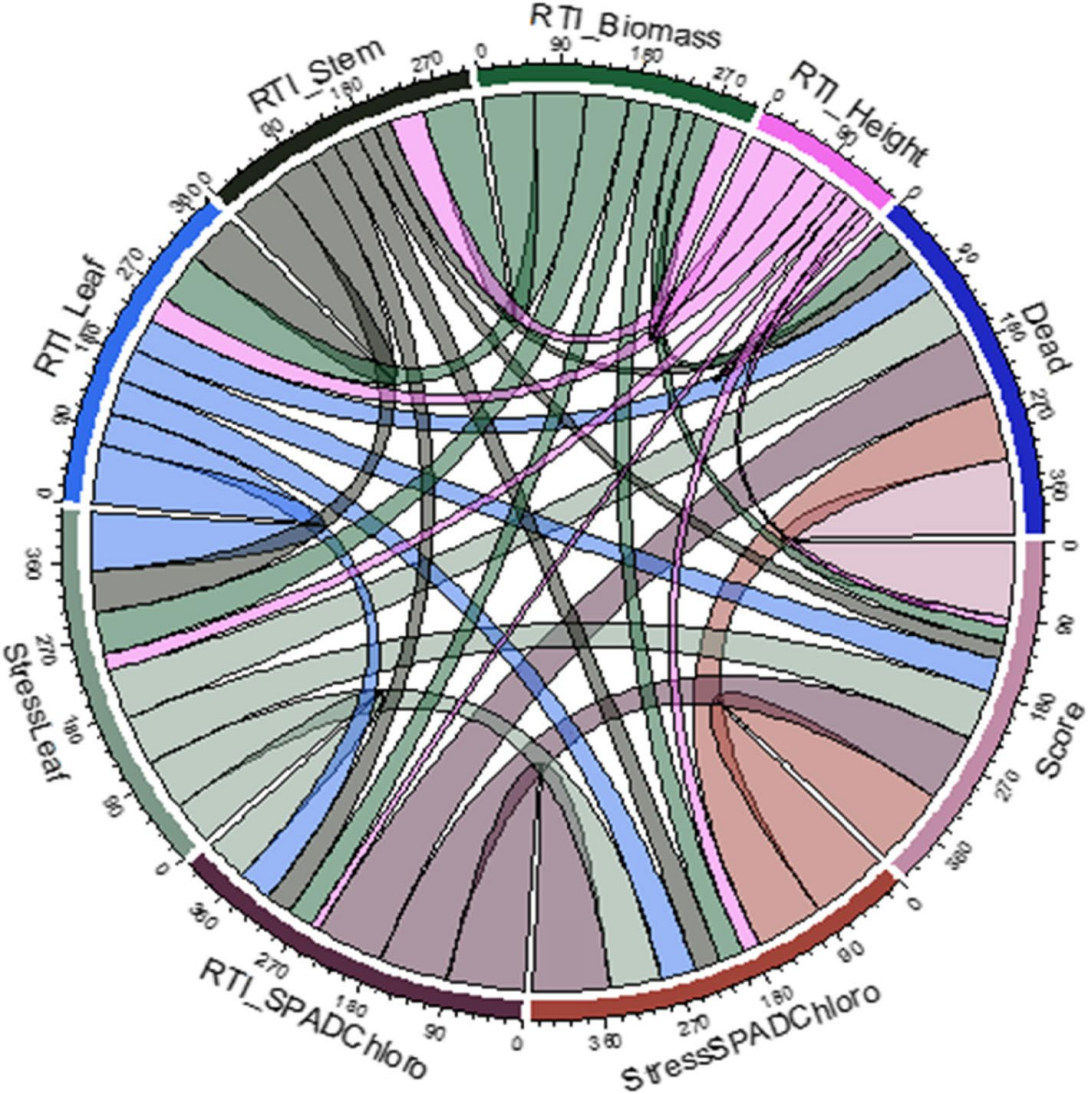
Manhattan plots for genome-wide association study (GWAS) corresponding to the average number of dead plants per pot (Dead), leaf injury score (Score), leaf SPAD chlorophyll under salt stress (S_Chloro), relative tolerance index for leaf SPAD chlorophyll (RTI_C), fresh leaf biomass under salt stress (S_Leaf), relative tolerance index for fresh leaf biomass (RTI_FL), relative tolerance for fresh stem biomass (RTI_FS), relative tolerance index for total above-ground fresh biomass (RTI_FB), and relative tolerance index for plant height (RTI_H). For each Manhattan plot, the x-axis represents the chromosome number and the y-axis indicates the –log_10_(p) where is the p-value corresponding to each SNP after running BLINK. The horizontal red line indicates the LOD threshold

A total of seven significant SNPs were identified to be associated with the average number of dead plants per pot (Table 4), among which three SNPs were located on chromosome 3 and four SNPs on chromosome 7 (Fig. 3). The three SNPs on chromosome 3 span a 48-kb region. The significant SNPs that were found on chromosome 7 were 2_25790 (LOD=4.1, MAF=13.6%), 2_07660 (LOD=3.7, MAF=11.6%), 2_02219 (LOD=3.7, MAF=11.6%), and 2_02220 (LOD=3.7, MAF=11.6%). The SNPs 2_07660, 2_02219, and 2_02220 were located within a 15-kb region of chromosome 7. One annotated gene was identified within the 20-bk region harboring each significant SNP. The annotated genes found within or in the vicinity of each SNP location encoded for a homeobox associated leucine zipper, xyloglucan:xyloglucosyl transferase, RNA helicase, leucine rich repeat, calcium-dependent protein kinase 32, typa-like translation elongation factor SVR3-related, and raffinose synthase/seed imbibition protein Sip1 (Table 4). A total of 2 SNPs were found to be significantly associated with leaf injury score. These SNPs were 2_13484 (LOD=3.6, MAF=29.3%) and 2_13485 (LOD=3.6, MAF=29.3%), and located at 25,524,675 bp and 25,525,542 bp on chromosome 1, respectively (Fig. 3). The annotated gene found in the vicinity of these SNPs was *Vigun01g093100.1*, which encodes for a Na^+^/Ca^2+^ K^+^ independent exchanger (Table 4).

**Table 4:** List of SNPs significantly associated with the traits evaluated under salt tolerance in a MAGIC cowpea population, chromosome and physical position (bp) of each SNP, LOD (-log10(p-value)), minor allele frequency MAF (%), annotated genes found within the 20-kb region flanking each significant SNP, and functional annotations for each gene ID. LOD threshold was greater or equal to 3.5. If no SNPs were above the threshold, the top 3 SNPs with the highest LOD were listed in below table. The BLINK model does not compute R_square, so no R_square information is provided

Traits	SNP	Chr	Position (bp)	LOD	MAF(%)	Gene_ID	Functional_annotation
DeadPlants	2_26528	3	47,346,498	4.1	35.1	*Vigun03g290500.1*	Homeobox associated leucine zipper
	2_05819	3	47,359,021	4.1	35.1	*Vigun03g290600.1*	Xyloglucan:xyloglucosyl transferase
	2_28348	3	47,394,698	3.7	35.7	*Vigun03g290800.1*	NA
	2_25790	7	1,969,327	4.1	13.6	*Vigun07g023000.1*	RNA helicase
						*Vigun07g023100.1*	Leucine Rich repeat
	2_07660	7	3,048,839	3.7	11.6	*Vigun07g032400.1*	Calcium-dependent protein kinase 32
						*Vigun07g032300.1*	Typa-like translation elongation factor SVR3-related
	2_02219	7	3,062,497	3.7	11.6	*Vigun07g032500.1*	Raffinose synthase or seed imbibition protein Sip1
	2_02220	7	3,063,296	3.7	11.6	*Vigun07g032500.1*	Raffinose synthase or seed imbibition protein Sip1
Score	2_13484	1	25,524,675	3.6	29.3	*Vigun01g093100.1*	Na+/Ca2+ K+ independent exchanger
	2_13485	1	25,525,542	3.6	29.3	*Vigun01g093100.1*	Na+/Ca2+ K+ independent exchanger
S_Chloro	2_14317	3	43,217,726	3.7	38.0	*Vigun03g263100.1*	Mitochondrial folate transporter/carrier
	2_33024	3	43,218,173	4.2	49.6	*Vigun03g263100.1*	Mitochondrial folate transporter/carrier
	2_45043	3	43,435,268	3.6	38.4	*Vigun03g264700.1*	NA
	2_15070	3	43,489,540	3.6	38.8	*Vigun03g265200.1*	Auxilin/cyclin G-associated kinase-related
	2_02054	3	43,739,483	3.9	48.8	*Vigun03g267000.1*	Clathrin coat assembly protein
						*Vigun03g267100.1*	Lysine methyltransferase
						*Vigun03g266900.1*	Phytoene dehydrogenase
	2_29692	3	43,757,044	3.9	48.8	*Vigun03g267200.1*	Retinaldehyde binding protein-related
	2_07148	3	43,786,460	3.7	49.2	*Vigun03g267400.1*	Succinate dehydrogenase flavoprotein subunit
	2_46677	3	44,031,642	3.6	48.8	NA	NA
	2_47326	3	44,089,702	3.6	48.8	NA	NA
	2_31683	3	44,242,654	3.6	48.8	*Vigun03g269900.1*	Protein DA1-related
	2_51323	3	44,389,344	3.7	49.6	*Vigun03g270400.1*	Cysteine-rich secretory protein family
						*Vigun03g270300.1*	NA
	2_20981	3	44,394,170	3.7	49.6	*Vigun03g270400.1*	Cysteine-rich secretory protein family
						*Vigun03g270300.1*	NA
	2_20980	3	44,394,695	3.7	49.6	*Vigun03g270400.1*	Cysteine-rich secretory protein family
						*Vigun03g270300.1*	NA
	2_51556	3	44,395,302	3.7	49.6	*Vigun03g270400.1*	Cysteine-rich secretory protein family
						*Vigun03g270300.1*	NA
	2_27478	3	44,562,081	3.7	48.8	*Vigun03g271300.1*	Vacuolar protein sorting-associated protein VPS13
	2_26528	3	47,346,498	4.1	35.1	*Vigun03g263000.1*	Alpha/beta hydrolase fold
	2_05819	3	47,359,021	4.1	35.1	*Vigun03g290600.1*	Xyloglucan:xyloglucosyl transferase
	2_28348	3	47,394,698	4.0	35.7	*Vigun03g290800.1*	NA
RTI_C	2_14317	3	43,217,726	3.5	38.0	*Vigun03g263100.1*	Mitochondrial folate transporter/carrier
	2_33024	3	43,218,173	4.6	49.6	*Vigun03g263100.1*	Mitochondrial folate transporter/carrier
						*Vigun03g263000.1*	Alpha/beta hydrolase fold-containing protein
	2_15070	3	43,489,540	3.6	38.8	*Vigun03g265200.1*	Auxilin/cyclin G-associated kinase-related
	2_02054	3	43,739,483	4.1	48.8	*Vigun03g267000.1*	Clathrin coat assembly protein
						*Vigun03g267100.1*	Lysine methyltransferase
						*Vigun03g266900.1*	Phytoene dehydrogenase
	2_29692	3	43,757,044	4.1	48.8	*Vigun03g267200.1*	Retinaldehyde binding protein-related
	2_07148	3	43,786,460	3.8	49.2	*Vigun03g267400.1*	Succinate dehydrogenase flavoprotein subunit
	2_46677	3	44,031,642	3.9	48.8	NA	NA
	2_47326	3	44,089,702	3.9	48.8	NA	NA
	2_31683	3	44,242,654	3.9	48.8	*Vigun03g269900.1*	Protein DA1-related
	2_51323	3	44,389,344	4.0	49.6	*Vigun03g270400.1*	Cysteine-rich secretory protein-related
						*Vigun03g270300.1*	NA
	2_20981	3	44,394,170	4.0	49.6	*Vigun03g270400.1*	Cysteine-rich secretory protein-related
						*Vigun03g270300.1*	NA
	2_20980	3	44,394,695	4.0	49.6	*Vigun03g270400.1*	Cysteine-rich secretory protein-related
						*Vigun03g270300.1*	NA
	2_51556	3	44,395,302	4.0	49.6	*Vigun03g270400.1*	Cysteine-rich secretory protein-related
						*Vigun03g270300.1*	NA
	2_27478	3	44,562,081	3.9	48.8	*Vigun03g271300.1*	Vacuolar protein sorting-associated protein VPS13
	2_26528	3	47,346,498	3.9	35.1	*Vigun03g290500.1*	Homeobox-leucine zipper protein HAT9
	2_05819	3	47,359,021	3.9	35.1	*Vigun03g290600.1*	Xyloglucan:xyloglucosyl transferase
	2_28348	3	47,394,698	3.8	35.7	*Vigun03g290800.1*	NA
S_Leaf	2_27478	3	44,562,081	3.0	48.8	*Vigun03g271300.1*	Na+/Ca2+ K+ independent exchanger
	2_28348	3	47,394,698	3.3	35.7	*Vigun03g290800.1*	NA
	2_50921	7	16,162,316	3.1	24.3	NA	NA
RTI_FL	2_27478	3	44,562,081	3.9	48.8	*Vigun03g271300.1*	Vacuolar protein sorting-associated protein VPS13
	2_28348	3	47,394,698	4.0	35.7	*Vigun03g290800.1*	NA
RTI_FS	2_20734	4	40,193,498	3.4	10.3	*Vigun04g178400.1*	Glycosyltransferase 8 domain-containing protein
						*Vigun04g178500.1*	NA
						*Vigun04g178300.1*	CCR4-not transcription complex related
	2_13286	4	40,198,028	3.4	10.3	*Vigun04g178400.1*	Glycosyltransferase 8 domain-containing protein
						*Vigun04g178500.1*	NA
	2_13285	4	40,198,314	3.4	10.3	*Vigun04g178400.1*	Glycosyltransferase 8 domain-containing protein
						*Vigun04g178500.1*	NA
	2_44170	4	40,238,551	3.4	10.3	*Vigun04g178900.1*	H+/oligopeptide symporter
						*Vigun04g178800.1*	NA
	2_47221	4	40,244,092	3.4	10.3	*Vigun04g178900.1*	H+/oligopeptide symporter
						*Vigun04g179000.1*	Zinc finger FYVE domain containing protein
RTI_FB	2_33574	5	579,544	3.6	7.0	*Vigun05g006800.1*	Mannose-6-phosphate isomerase
						*Vigun05g006700.1*	Alpha/beta hydrolase fold-containing protein
						*Vigun05g006600.1*	NA
						*Vigun05g006500.1*	Neoxanthin biosynthesis
RTI_H	2_26489	3	20,639,699	4.2	28.1	*Vigun03g171400.1*	O-methyltransferase-related
	1_0247	3	20,639,954	4.2	28.1	*Vigun03g171400.1*	O-methyltransferase-related
	2_04756	3	20,640,004	4.2	28.1	*Vigun03g171400.1*	O-methyltransferase-related
	2_34159	3	21,168,375	4.2	28.9	*Vigun03g172900.1*	Protein transport protein Sect. 23
						*Vigun03g173100.1*	Peptidyl-prolyl cis-trans isomerase
	2_34562	3	21,184,999	4.2	29.0	*Vigun03g173200.1*	Cystatin-C
	2_00955	3	21,195,566	4.2	28.9	*Vigun03g173300.1*	Phospholipases
	2_52154	3	21,311,445	4.2	28.9	*Vigun03g173800.1*	Dolichol-phosphate mannosyltransferase
	2_15515	3	21,332,934	4.2	28.9	*Vigun03g174100.1*	IQ-domain 9 protein
						*Vigun03g174000.1*	Mutt-nudix-related
						*Vigun03g173900.1*	Magnesium chelatase subunit I
	2_06057	3	21,415,465	4.2	28.9	*Vigun03g174200.1*	Ionotropic glutamate receptor
						*Vigun03g174300.1*	Apoptosis inhibitor 5
	2_03596	3	21,479,991	4.2	28.9	*Vigun03g174500.1*	NA
						*Vigun03g174400.1*	Peroxidase 19
	2_45312	3	21,500,420	4.2	28.9	*Vigun03g174600.1*	Triacylglycerol degradation
	2_39953	3	21,742,682	3.8	28.9	NA	NA
	2_30884	3	21,777,011	3.8	28.9	*Vigun03g175900.1*	Cytochrome P450
	2_37604	3	21,810,301	3.8	28.9	NA	NA
	2_32781	3	21,841,991	3.8	28.9	*Vigun03g176100.1*	Microfibril-associated protein
	2_25800	3	21,872,524	3.8	28.9	*Vigun03g176300.1*	Suberin monomers biosynthesis
	2_14391	3	21,913,428	3.8	28.9	*Vigun03g176400.1*	Homoserine dehydrogenase
	2_14392	3	21,914,412	3.8	28.9	*Vigun03g176400.1*	Homoserine dehydrogenase
	2_54159	3	22,010,385	3.8	28.9	*Vigun03g177000.1*	Beta-galactosidase 9
						*Vigun03g177100.1*	Beta-galactosidase 9
	2_52111	3	22,014,192	3.8	28.9	*Vigun03g177000.1*	Beta-galactosidase 9
						*Vigun03g177100.1*	Beta-galactosidase 9
	2_47286	3	22,025,277	3.8	28.9	*Vigun03g177100.1*	Beta-galactosidase 9
	2_49598	3	23,926,152	3.8	28.9	NA	NA
	2_15529	3	24,031,773	3.8	28.9	*Vigun03g184300.1*	NA

A strong candidate 4.2-Mb region of chromosome 3 was associated with the leaf SPAD chlorophyll under salt stress (Fig. 3), flanked by 18 significant SNPs (Table 4). Of these, 2_33024 (LOD=4.2, MAF=49.6%), 2_26528 (LOD=4.1, MAF=35.1%), 2_05819 (LOD=4.1, MAF=35.1%), 2_28348 (LOD=4.0, MAF=35.7%), 2_02054 (LOD=3.9, MAF=48.8%), and 2_29692 (LOD=3.9, MAF=48.8%) had the highest LOD. At least one annotated gene was identified in the vicinity of each significant SNP except for the SNPs 2_46677 and 2_47326. The candidate genes encode for various proteins such as mitochondrial folate transporter/carrier, auxilin/cyclin g-associated kinase-related, clathrin coat assembly protein, phytoene dehydrogenase, retinaldehyde binding protein-related, succinate dehydrogenase flavoprotein subunit, protein Da1-related, cysteine-rich secretory protein family, vacuolar protein sorting-associated protein VPS13, alpha/beta hydrolase fold, and xyloglucan:xyloglucosyl transferase (Table 4).

GWAS for RTI_C identified a total of 17 significant SNPs (Table 4). These SNPs were the ones that were associated with leaf SPAD chlorophyll under salt stress and were located within the 4.2-Mb genomic region of chromosome 3 (Fig. 3), suggesting a high likelihood of QTL(s) affecting salt tolerance based on leaf SPAD chlorophyll in this genomic region. For fresh leaf biomass under salt stress, no SNPs were above the declared threshold (LOD ≥ 3.5). The top 3 SNPs with the highest LOD for fresh leaf biomass were 2_27478 (LOD=3.0, MAF=48.8%), 2_28348 (LOD=3.3, MAF=35.7%), and 2_50921 (LOD=3.1, MAF=24.3%). The SNPs 2_27478 and 2_28348 were within the candidate region associated with both leaf SPAD chlorophyll content and RTI_C, indicating that there could be a common pathway for salt tolerance based on fresh leaf biomass under salt stress, leaf SPAD chlorophyll under salt stress, and RTI_C.

Two SNPs were found to be significantly associated with RTI_FL (Fig. 3). These SNPs were also identified to be associated with fresh leaf biomass under salt stress, RTI_C, and leaf SPAD chlorophyll under salt stress (Table 4). While no SNPs were above the chosen LOD threshold (3.5) for RTI_FS, those with marginal significance include 2_20734 (LOD=3.4, MAF=10.3%), 2_13286 (LOD=3.4, MAF=10.3%), 2_13285 (LOD=3.4, MAF=10.3%), 2_44170 (LOD=3.4, MAF=10.3%), and 2_47221 (LOD=3.4, MAF=10.3%). These SNPs were located with a 50.6-kb region of chromosome 4. A total of 6 annotated genes were found in the vicinity of these SNPs. These genes encode for a glycosyltransferase 8 domain-containing protein, ccr4-not transcription complex related, H+/oligopeptide symporter, and zinc finger FYVE domain containing protein.

One SNP, 2_33574, was significantly associated with RTI_FB (Fig. 3). This SNP was located at 579,544 bp on chromosome 5 (Table 4). The annotated genes found in the vicinity of this SNP were *Vigun05g006800.1*, *Vigun05g006700.1*, *Vigun05g006600.1*, and *Vigun05g006500.1*. No functional annotations were found for *Vigun05g006600.1*. Functional annotations for *Vigun05g006800.1*, *Vigun05g006700.1*, and *Vigun05g006500.1* were Mannose-6-phosphate isomerase, alpha/beta hydrolase fold-containing protein, and neoxanthin biosynthesis, respectively. GWAS suggested a strong candidate locus associated with RTI_H) (Fig. 3). This genomic region harbored a total of 23 significant SNPs and were mapped on a 3.4-Mb region of chromosome 3 (Table 4). The significant SNPs with the highest LOD were 2_26489 (LOD=4.2, MAF=28.1%), 1_0247 (LOD=4.2, MAF=28.1%), 2_04756 (LOD=4.2, MAF=28.1%), 2_34159 (LOD=4.2, MAF=28.9%), 2_34562 (LOD=4.2, MAF=29.0%), 2_00955 (LOD=4.2, MAF=28.9%), 2_52154 (LOD=4.2, MAF=28.9%), 2_15515 (LOD=4.2, MAF=28.9%), 2_06057 (LOD=4.2, MAF=28.9%), 2_03596 (LOD=4.2, MAF=28.9%), and 2_45312 (LOD=4.2, MAF=28.9%). A total of 27 annotated genes were identified in the vicinity of the significant SNPs associated with RTI_H (Table 4). Functional annotations associated with the candidate genes were O-methyltransferase-related, protein transport protein Sect. 23, peptidyl-prolyl cis-trans isomerase, cystatin-C, phospholipases, dolichol-phosphate mannosyltransferase, IQ-domain 9 protein, mutt-nudix-related, magnesium chelatase subunit I, ionotropic glutamate receptor, apoptosis inhibitor 5, peroxidase 19, triacylglycerol degradation, cytochrome P450, microfibril-associated protein, suberin monomers biosynthesis, homoserine dehydrogenase, and beta-galactosidase 9 (Table 4).

## Overlapping SNPs between traits

**Fig. 4:**
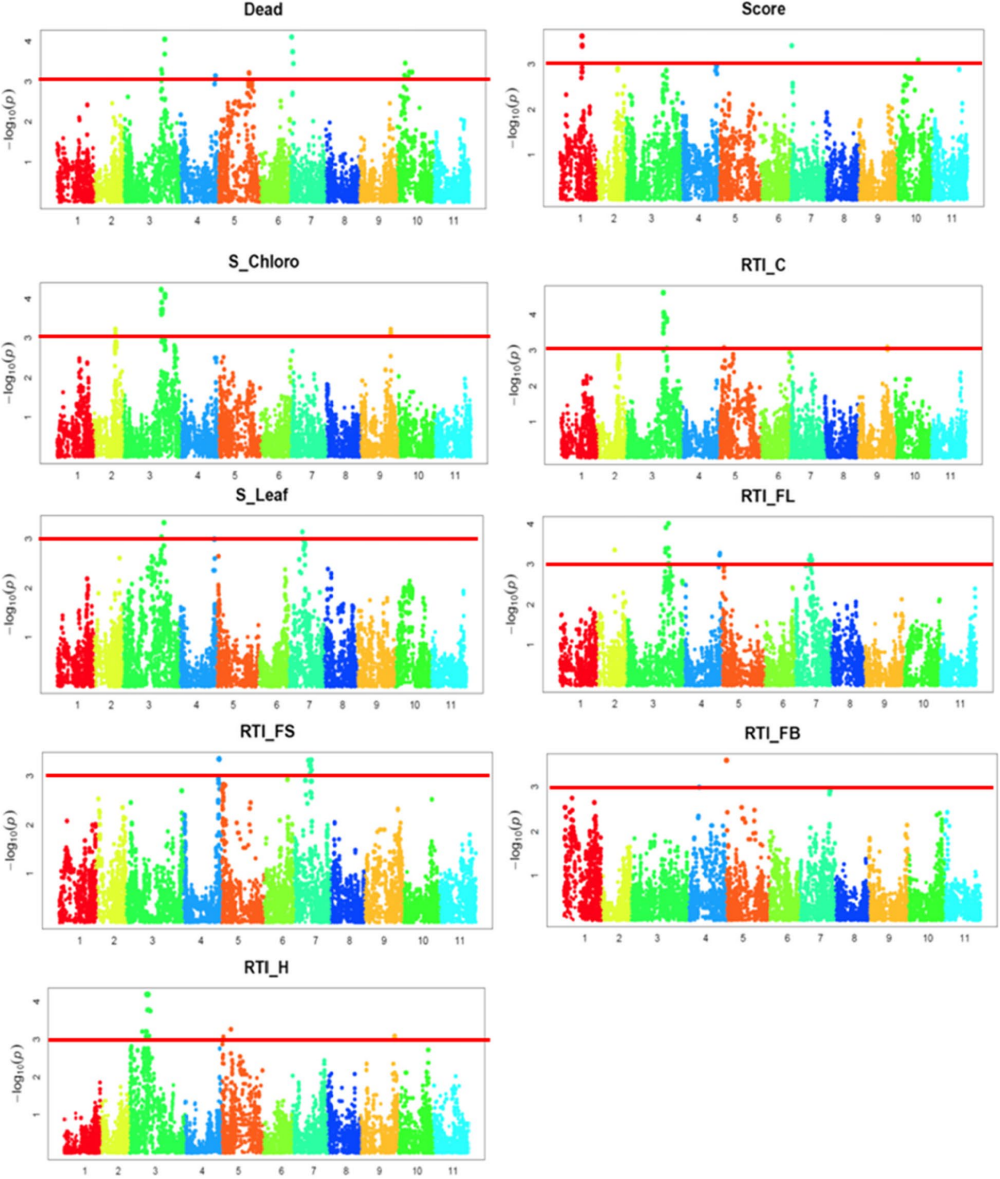
Venn diagram showing the overlapping significant SNP markers between the average number of dead plants per pot (Dead), leaf SPAD chlorophyll under salt treatment (S_C), relative tolerance index for leaf SPAD chlorophyll (RTI_Chloro), and relative tolerance index for plant height (RTI_H). Venn diagrams were established using the online software program that is accessible at http://jvenn.toulouse.inra.fr/app/example.html

Common SNP markers were identified between the traits evaluated for salt tolerance in this MAGIC cowpea population. Out of the SNP markers associated with the average number of dead plants per pot, a total of 3 were found to be associated with both leaf SPAD chlorophyll under salt treatment (S_Chloro) and relative tolerance index for leaf SPAD chlorophyll (RTI_C) (Fig. 4), indicating that there could be a common pathway for salt tolerance based on these traits. A total of 14 significant SNPs were overlapping between S_Chloro and RTI_C (Fig. 4). Interestingly, none of the significant SNP markers associated with relative tolerance index for plant height (RTI_H) overlapped with any SNP markers associated with other traits (Fig. 4), suggesting that the mechanism for salt tolerance based on RTI_H could be independent. Using a Venn diagram with more than 5 sets would be difficult to visualize, so the Venn diagram (Fig. 4) did not include the data for leaf injury score (Score), relative tolerance index for fresh stem biomass (RTI_FS), relative tolerance index for the total above-ground fresh biomass (RTI_FB), fresh leaf biomass under salt stress (S_Leaf), and relative tolerance index for fresh leaf biomass (RTI_FL). None of the SNP makers associated with Score overlapped with any SNP makers associated with other traits (Table 4). Similar results were found for RTI_FS and RTI_FB. One SNP associated with S_Leaf, 2_28348, overlapped with one SNP associated with RTI_FL, S_Chloro, Dead, and RTI_C (Table 4). The SNP 2_27478, associated with S_Leaf, was also associated with RTI_C, S_Chloro, and RTI_FL (Table 4). These results indicated that there could be a common pathway for salt tolerance between S_Leaf, RTI_FL, S_Chloro, Dead, and RTI_C.

## Epistatic interaction analysis

A total of 513,489,081 possible pairwise interactions were tested using PLINK v1.07 for each trait. Of these, a total of 949, 264, 161, 272, 413, 269, 1323, 395, and 341 pairwise interactions for the average number of dead plants per pot, leaf injury score, S_Chloro, RTI_C, S_Leaf, RTI_FL, RTI_FS, RTI_FB, and RTI_H, respectively, were significant based on our chosen threshold (p-value ≤ 10^−6^).

**Fig. 5:**
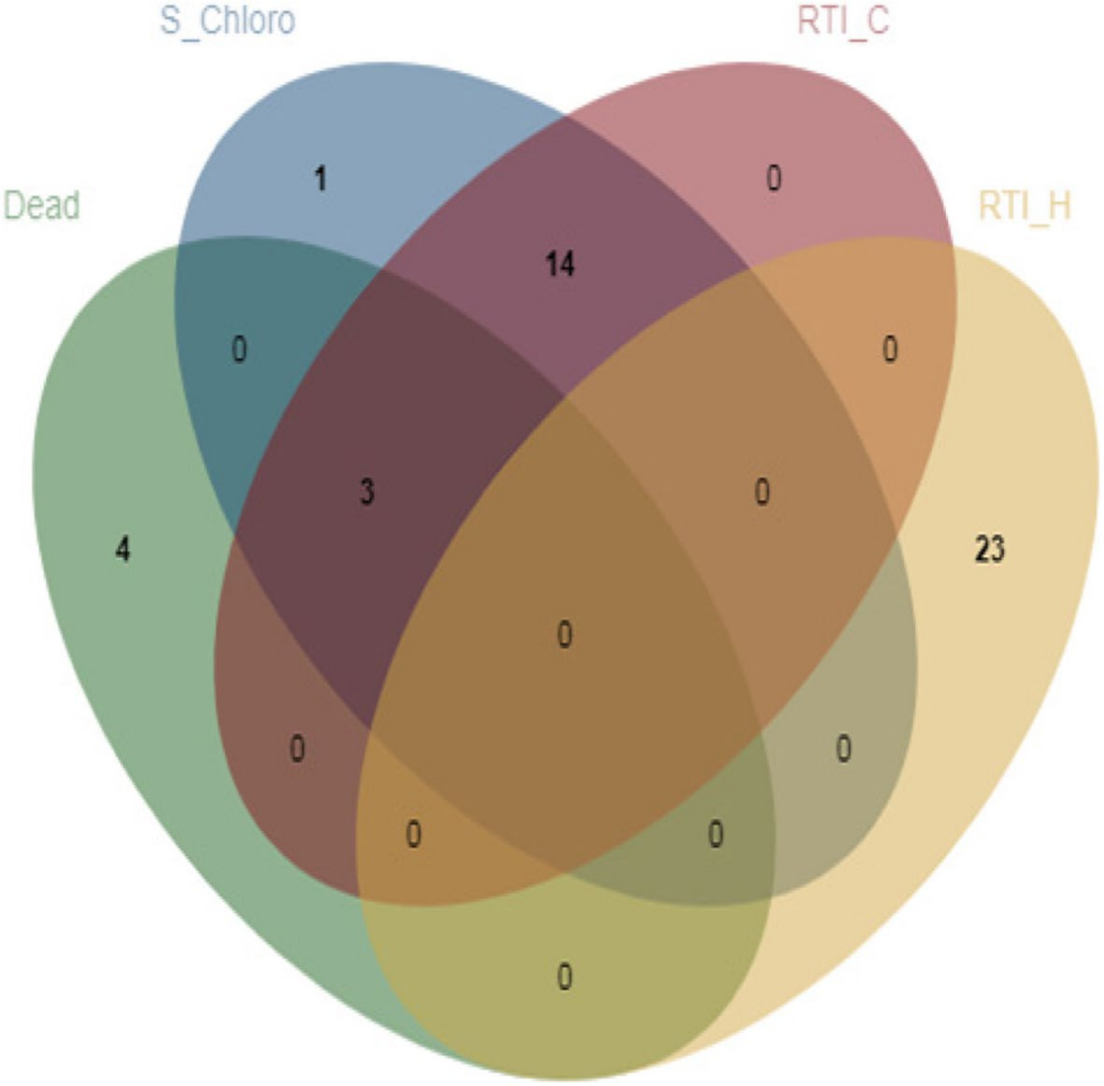
Circos plots showing the significant pairwise epistatic interactions between SNPs. On each circos plot, the outermost layer represents the 11 chromosomes of cowpea and the length of each segment is proportional to the length of each chromosome. The innermost layer displays the SNPs used for conducting GWAS and each black dot represents one SNP. The width of the innermost layer is proportional to the LOD values of each SNP. The further from the center the black dot is, the higher the LOD is. Links within each circos plot show a significant epistatic interaction between two SNPs. Since the resolution of the chromosomal length is in Mb (outermost layer), two closely located pairs of pairwise epistatic interactions can be cofounded in the above figure, so the number of links might not reflect the actual number of pairwise epistatic interactions. **A** Average number of dead plants per pot, **B** Salt injury score, **C** leaf SPAD chlorophyll under salt treatment, **D** relative tolerance index for leaf SPAD chlorophyll, and **E** fresh leaf biomass under salt stress

All pairwise epistatic interactions found for the average number of dead plants per pot were between chromosomes with chromosomes 9 and 11 having the highest number of significant epistasis (Fig. 5 A). However, these epistasis-rich regions had SNPs with low LOD values. The genomic region of chromosome 7 that harbors some of the significant SNP markers associated with the average number of dead plants per pot was in epistasis with some SNPs found at the beginning of chromosome 8 (Fig. 5 A). No significant interaction was identified between SNPs located within the two candidate loci (one on chromosome 3 and the other on chromosome 7) associated with the average number of dead plants per pot. Similar results were found for leaf injury score where no epistatic interactions were identified between the significant SNPs associated this trait (Fig. 5B). The chromosomes with the highest number of epistatic interactions were chromosome 3 and chromosome 8 (Fig. 5B). Interestingly, most of significant epistatic interactions for leaf injury score appeared to be located towards both ends of the chromosome as shown in Fig. 5B. The epistasis analysis results for S_Chloro were particularly complex in which significant SNP markers associated with this trait, which were located on chromosome 3, were in epistatic interaction with SNPs located on chromosomes 2, 8, and 11 (Fig. 5 C).

**Fig. 6:**
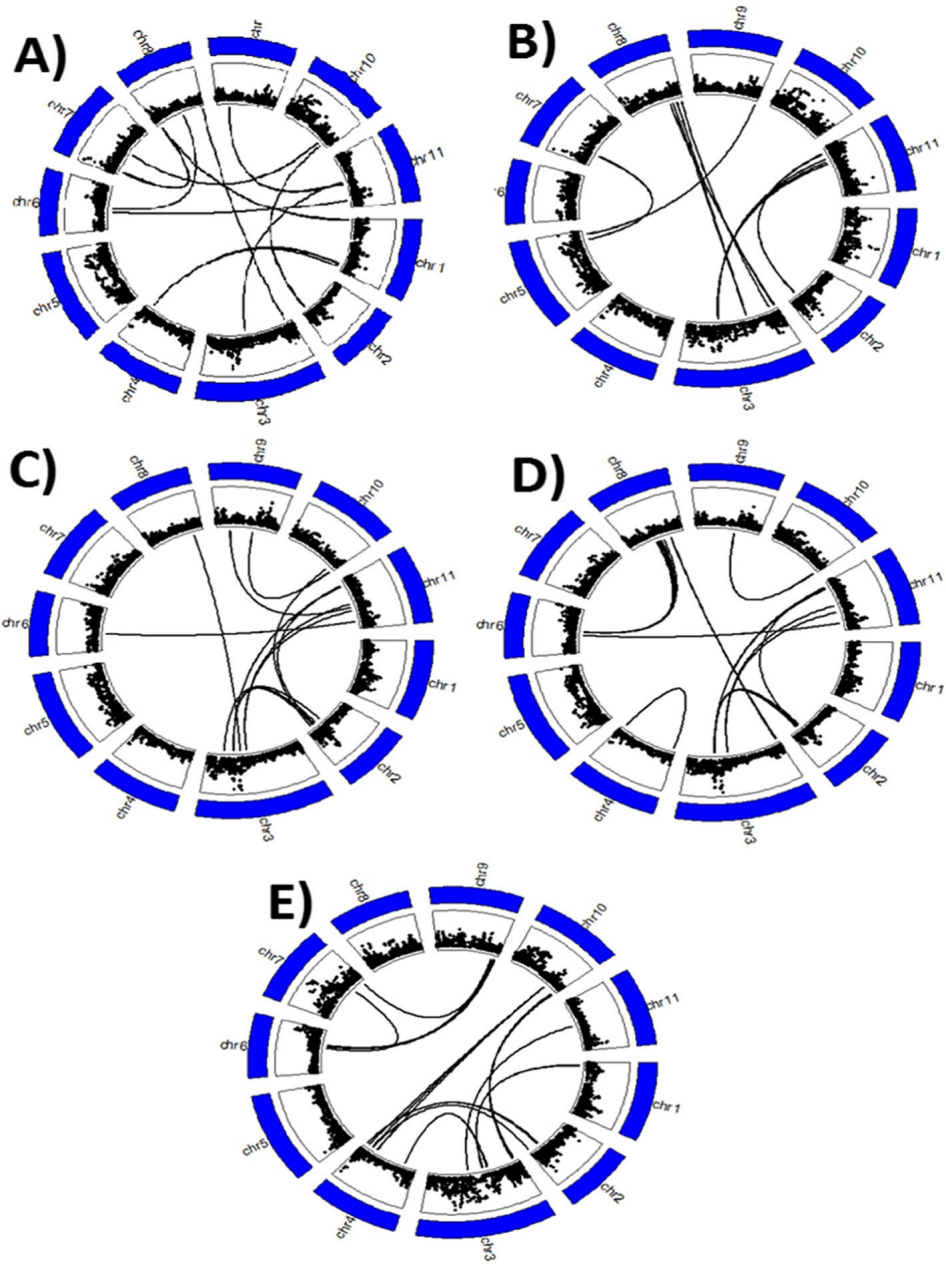
Circos plots showing the significant pairwise epistatic interactions between SNPs. On each circos plot, the outermost layer represents the 11 chromosomes of cowpea and the length of each segment is proportional to the length of each chromosome. The innermost layer displays the SNPs used for conducting GWAS and each black dot represents one SNP. The width of the innermost layer is proportional to the LOD values of each SNP. The further from the center the black dot is, the higher the LOD is. Links within each circos plot show a significant epistatic interaction between two SNPs. Since the resolution of the chromosomal length is in Mb (outermost layer), two closely located pairs of pairwise epistatic interactions can be cofounded in the above figure, so the number of links might not reflect the actual number of pairwise epistatic interactions. **A** Relative tolerance index for fresh leaf biomass, **B** relative tolerance index for fresh stem biomass, **C** relative tolerance index for total above-ground fresh biomass, and **D** relative tolerance index for plant height

Results indicated a within-chromosome epistatic interaction (chromosome 4) for RTI_C (Fig. 5D). The pattern of epistasis for RTI_C was very similar to that of S_Chloro (Fig. 5 C and D), which was expected since these traits were highly correlated (Table 3). The chromosomes with the highest number of significant epistasis for S_Leaf were 3 and 4 (Fig. 5E). None of the significant SNP markers associated with S_Leaf were in epistasis with any other SNPs. For RTI_FL, chromosomes 6 and 7 had the highest number of significant epistatic interactions and a within-chromosome epistasis was found on chromosome 7 (Fig. 6 A). The significant SNP markers associated with RTI_FL and found on chromosome 3 were not in epistatic interaction with any other SNPs (Fig. 6 A).

Epistatic interactions were also identified for RTI_FS. The chromosomes with the highest epistatic interaction were 1, 6, and 11 (Fig. 6B). The significant SNPs associated with RTI_FS and located on chromosome 4 were in epistatic interaction with some low-LOD SNPs on chromosome 7 (Fig. 6B). However, the other candidate locus containing significant SNPs and located on chromosome 7 was not in epistatic interaction with any genomic regions. RTI_FB had the highest number of significant epistatic interactions among all traits evaluated for salt tolerance in this study. The chromosomes with the highest number of significant epistatic interactions for RTI_FB were 3, 6, and 10 (Fig. 6 C). The significant SNP markers associated with RTI_FB and mapped on chromosome 5 were in epistatic interaction with some low-LOD SNPs on chromosome 6. No within-chromosome epistatic interactions were identified for RTI_FB. The significant SNP markers associated with RTI_H and located on chromosome 3 were not in epistatic interaction with any other SNPs as shown in Fig. 6D. One within-chromosome epistatic interaction was found on chromosome 7.

## Discussions

The large phenotypic variation and heritabilities observed in 234 MAGIC lines and 8 founder parents suggest that this population would be sufficient for salt tolerance genetic studies. To the best of our knowledge, this study was one of the earliest reports investigating salt tolerance based on a MAGIC population in cowpea. In addition, the population size for this study was larger than those used in previous reports investigating cowpea salt tolerance [21, 23].

GWAS identified significant SNP markers associated with various traits evaluated under salt stress in this MAGIC cowpea population, expanding the list of SNP markers associated with other important traits that were previously reported for cowpea [7, 33–35]. The earliest study for salt tolerance also identified several SNPs associated with salt tolerance in cowpea, including Scaffold87490_622, Scaffold87490_630, C35017374_128, Scaffold93827_270, Scaffold68489_600, Scaffold87490_633, Scaffold87490_640, Scaffold82042_3387, C35069468_1916, and Scaffold93942_1089 [23]. These SNPs were identified by conducting GWAS based on a diversity panel of 155 cowpea lines and 1049 SNPs that were postulated from genotyping-by-sequencing. In the present study, we performed GWAS based on a larger panel genotyped with a large number of SNPs. In addition, most of the SNP markers identified in this study were within or in the vicinity of annotated genes whose functional annotations involved salt tolerance mechanisms, which provides confidence to our findings.

Various candidate genes encoding for proteins with possible functions in salt tolerance mechanisms have been identified. The candidate gene (*Vigun01g093100.1*) revealed a relationship between Na^+^/Ca2^+^ K^+^ independent exchanger and salt tolerance in cowpea. The involvement of Na^+^/Ca2^+^ K^+^ independent exchanger in salt tolerance has been well described in other species such as tomato and soybean [36]. Therefore, the SNP markers (2_13484 and 2_13485) found in the vicinity of this gene could be reliably used for screening salt tolerance in cowpea. In addition, a H+/oligopeptide symporter has been shown to be associated with Cl- dynamic under salt stress in soybean [37]. These results suggested that a common salt tolerance mechanism pathway could exist between soybean and cowpea. Calcium-dependent protein kinases have also been identified to be associated with salt tolerance based on our data. Another study showed that calcium-dependent protein kinases are important in regulating responses to salt stress in cotton [38]. These proteins play a role in stress signaling. Transcripts encoding for calcium-dependent protein kinases were also reported to be induced at early stage of salt stress in cotton [38]. These findings suggested that similar salt tolerance mechanism could exist in cowpea. Our candidate gene search based on trait-associated SNPs supports the involvement of vacuolar proteins in salt tolerance in cowpea. Vacuolar proteins are critical in maintaining the trans-Golgi network (TGN) during salt tolerance [39]. In addition, *Vigun03g290600.1* has been reported to encode for xyloglucan:xyloglucosyl transferase that is induced upon salt stress in *Arabidopsis thaliana* [40]. Despite of the possible relationship existing between functional annotations of the candidate genes identified in this study and salt tolerance mechanism, further studies including transcriptomic analysis would be required to validate these genes.

Soil salinity has been shown to be a growing threat to agriculture worldwide [9]. Cowpea can be significantly impaired by soil salinity [8]. In this investigation we reported the variation of salt tolerance in a MAGIC cowpea population and identified salt-tolerant MAGIC lines. This MAGIC population has been registered [28], but lacking information on tolerance to salt stress. Therefore, our results complement the previously collected information on this MAGIC population and helps further increase its usefulness in cowpea breeding.

## Conclusions

Our findings contribute to providing useful information for basic and applied research in genetic improvement to address soil salinity. Large variation in salt tolerance among the cowpea MAGIC lines has been identified. The salt-tolerant lines could be used as donor parents in breeding for salt tolerance in cowpea. In addition, a large number of cowpea SNP markers were found within or in the vicinity of genes that were reported to be directly involved in salt tolerance in other crops. Therefore, these SNPs can provide a framework for map-based cloning and designing gene-based markers in cowpea and other crops based on synteny. Breeding for salt tolerance via marker-assisted selection (MAS) would also be possible. However, additional studies would be needed to validate the candidate genes identified in this study.

## Materials and methods

### Population development and genotyping

The MAGIC population development was previously described [28]. In brief, the first crosses between eight founder parents (IT89KD-288, IT84S-2049, CB27, IT82E-18, Suvita 2, IT00K-1263, IT84S-2246, and IT93K-503-1) were conducted in 2011. These parents were elite cultivars and breeding lines from Sub-Saharan Africa and the United States [41–46]. Greenhouse-grown seeds of 305 F_8:10_ RIL lines and eight founders were obtained from University of California, Riverside (UCR). Ten seeds per line were hand-planted in a 5-foot-long row at the research station of the University of Arkansas, Fayetteville during the summer of 2018. Some lines were not able to flower due to photoperiodism under the Arkansas climate. A total of 234 MAGIC lines and eight parents were harvested. Seeds from each row were bulked for use in the salt tolerance evaluation.

The MAGIC population and the founders were genotyped using a total of 51,128 SNPs obtained from the Illumina Cowpea Consortium Array [47]. The SNP data as well as genetic diversity analysis of this population were previously reported [28]. After filtering, a total of 32,047 SNPs (missing data<10%, heterozygosity<10%, and minor allele frequency>5%) were used for further analysis.

### Growth conditions and experimental design

**Fig. 7:**
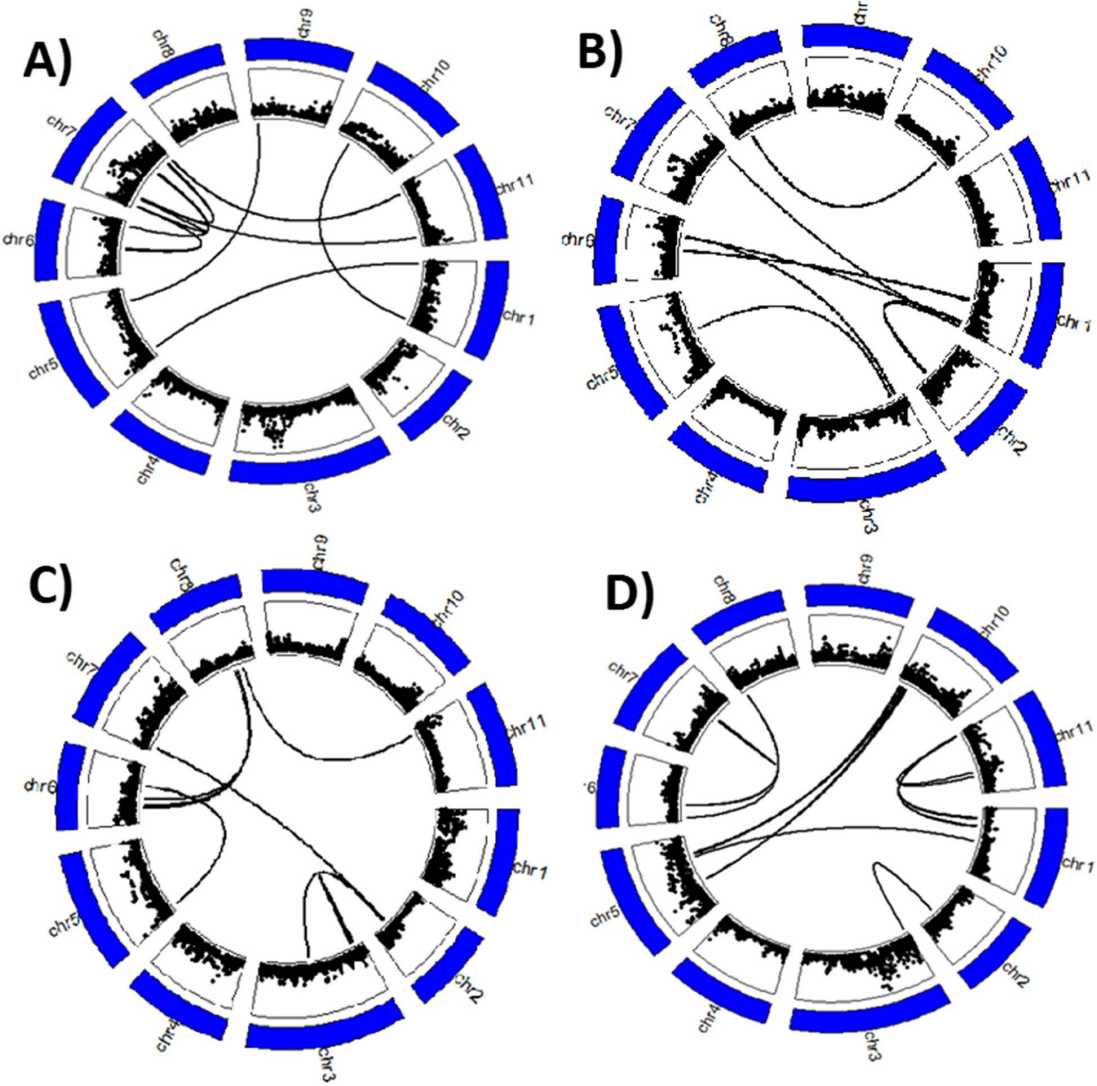
Greenhouse experiment for salt tolerance evaluation on a MAGIC cowpea population. (R): the resistant control (09-529), (S): the susceptible control (PI252774)

Salt tolerance evaluation was conducted using a previously described methodology [48]. The experiment was carried out in the greenhouse of Harry R. Rosen Alternative Pest Control of the University of Arkansas, Fayetteville where the average temperature was 26 °C/21 °C (day/light) and the daylight length was 14 h (Fig. 7). Cowpea seeds were sown in pots previously filled up with 100 g Sunshine Natural & Organic (Agawam, MA). At the bottom of each pot was designed holes to prevent water from being stored during irrigation, which could lead to plant root asphyxia. In addition, paper towels were established at the bottom of each pot to prevent soil from leaking during irrigation. In each pot, a total of 8 seeds were sown and thinned to a total of 4 vigorous and uniform plants at one week after emergence. Plants were fertilized weekly by applying a solution of 50 mL of Miracle-Gro fertilizers (Scotts Miracle-Gro, Detroit, MI) to each pot.

The experiment was a randomized complete block design (RCBD) with two blocks and two replications within each block. Pots containing cowpea plants were placed on rectangular plastic trays to make the irrigation process convenient. For each line per replication, one pot was used as control receiving deionized water during irrigation, and the other pot receiving a salt treatment, so there were one salt-treated and one non-salt-treated pot for each replication.

The salt treatment (NaCl) started when the first trifoliate leaf began to expand (V1 stage) [49]. Salt concentration was 200 mM NaCl as previously suggested [23, 50–52]. Irrigation was applied to each tray containing 12 pots with either deionized water or salt solution. Irrigation was maintained in such a way that two-third of pot height was soaked with the treatment solution.

One salt-tolerant cowpea genotype (‘09-529’) and one salt-susceptible cowpea genotype (PI255774) were also included as checks [21, 23]. Two subsets of the extremes, including ten most salt-tolerant and ten most salt-susceptible lines, were repeated at the end of the experiments.

### In vivo chlorophyll measurement

Leaf chlorophyll was measured using a SPAD-502 Plus (Spectrum Technologies, Inc., Plainfield, IL). Measurements were achieved at one day prior to salt treatment and when the susceptible controls were completely dead, which was about 14 days after the first salt stress. For each plant, chlorophyll measurement was conducted three times on both trifoliate and unifoliate times, respectively, and the average read was recorded and analyzed. Measurements were done on three different positions on the leaf surface in order to limit the edge effect [21, 23]. Data were taken from all plants under salt stress and non-salt stress conditions.

### Plant fresh above-ground parameter

Plant height was recorded for each cowpea seedling at one day before the salt treatment began and when the susceptible controls were dead, indicative of the end of plant growth in the susceptible genotypes [23]. Data on both fresh leaf and fresh stem biomass from each plant were also taken. The above-ground fresh biomass corresponded to the sum of fresh leaf and stem weights.

### Leaf injury scoring

Leaf injury score has been successfully used as a reliable parameter for screening salt tolerance at the seedling stage in cowpea [23]. It has been shown to be highly correlated with Na^+^ and Cl^−^ contents in leaves [53], and can accurately assess salt tolerance/susceptibility when leaf ion extraction is costly [23, 53]. Leaf injury scoring was conducted when the susceptible controls were completely dead, using a previously established scale (1 = healthy plants, 2 = sign of leaf chlorosis, 3 = expansion of chlorosis on leaf surface, 4 = totally chlorotic leaf, 5 = first sign of necrosis, 6 = expansion of necrosis on leaf surface, and 7 = completely dead plants) (Ravelombola et al. 2017).

### Phenotypic data analysis

Relative tolerance indexes (RTIs) for chlorophyll, plant height, fresh leaf biomass, fresh stem biomass, and total fresh above-ground biomass were used to assess the impact of salt stress relative to the non-salt stress condition. RTIs were calculated as following [23, 54].

- RTI_chlorophyll (RTI_C) = (Y_c_S_/Y_c_NS_) * 100.
- RTI_plant_height (RTI_H) = (Y_h_S_/Y_h_NC_) * 100.
- RTI_fresh_leaf_biomass (RTI_FL) = (Y_l_S_/Y_l_NS_) * 100.
- RTI_fresh_stem_biomass (RTI_FS) = (Y_s_S_/Y_s_NS_) X 100.
- RTI_total_above_fresh_ground_biomass (RTI_FB) = (Y_b_S_/Y_b_NS_) * 100.

Where Y_c_S_: the chlorophyll content under salt stress, Y_c_NS_:the chlorophyll content under non-salt stress, Y_h_S_: the plant height under salt stress, Y_h_NC_: the plant height under non salt stress, Y_l_S_: the fresh leaf biomass under salt stress, Y_l_NS_: the fresh leaf biomass under non-salt stress, Y_s_S_: the fresh stem biomass under salt stress, Y_s_NS_: the fresh stem biomass under non-salt stress, Y_b_S_: the total fresh above ground biomass under salt stress, and Y_b_NS_: the total fresh above ground biomass under non-salt stress.

Data distribution was visualized using the MASS package of the R® 3.6.1. version. The analysis of variance (ANOVA) was done using PROC MIXED of SAS® 9.4 (SAS Institute Inc., Cary, NC). Mean separation was conducted using a protected least significant difference (LSD) procedure at α = 0.05. LSD procedure was defined as LSD=t_α/2_√2MSError/n, with t_α/2_ being the critical value from the t-table and having a degree of freedom [df(SSError)] corresponding to the difference between the number of observations and the number of replications, and n being the number of replications. The ANOVA model was as follows.

$${\mathrm Y}_{\mathrm i(\mathrm j)\mathrm k}\:=\:\mu\:+\:{\mathrm T}_{\mathrm j}\;+\;{\mathrm G}_{\mathrm k}\;+\;{\mathrm R}_{\mathrm i(\mathrm j)}+\;{\mathrm{TG}}_{\mathrm{jk}}\:+\:{\mathrm\varepsilon}_{\mathrm i(\mathrm j)\mathrm k}\;\mathrm{where}\;\mathrm i\:=\:1,2,\;\mathrm j\:=\:1,2,\;\mathrm{and}\;\mathrm k\:=\:1\dots234$$

with µ being the overall mean, Y_i(j)k_ being the response from the k^th^ genotype (G_k_) (fixed effect) at the i^th^ replication (R_i(j)_), which was nested under the j^th^ run (block) (T_j_)(fixed effect), and TG_jk_ being the interaction effect between the k^th^ genotype (G_k_) and the j^th^ run (block) (T_j_).

The broad sense heritability (*H*) was estimated using the following formula [55].

$$\mathrm H\:=\:\mathrm\sigma_{\mathrm G}^2/\left[\mathrm\sigma_{\mathrm G}^2\;+\left(\left(\mathrm\sigma_{\mathrm{GXR}}^2\right)/{\mathrm n}_{\mathrm b}\right)\;+\;\left(\left(\mathrm\sigma_{\mathrm e}^2\right)/\left({\mathrm n}_{\mathrm b}\ast{\mathrm n}_{\mathrm r}\right)\right)\right]$$

with σ^2^_G_ being the total genetic variance, σ^2^_GXR_ being the Genotype X Run variance, σ^2^_e_ being the residual variance, n_b_ being the number of runs, and n_r_ being the number of replications. The estimates for σ^2^_G_ and σ^2^_GXR_ were [EMS(G)-EMS(GXB)]/ n_b_*n_r_ and [EMS(GXB)-Var(Residual)]/n_r_. EMS(G), EMS(GXB), and Var(Residual) were obtained from the ANOVA table.

Pearson’s correlation coefficients between the average number of dead plants per pot, average leaf injury score, fresh leaf biomass under salt stress, SPAD chlorophyll content under salt stress, RTI_C, RTI_H, RTI_FL, RTI_FS, and RTI_FB were calculated using the R® 3.6.1. version. A chord diagram was used in order to better visualize the pairwise correlation between traits. Chord diagram was established in R® v.3.6.1 using the package ‘circlize’ [56].

### Genome-wide association study (GWAS)

GWAS was conducted using a Bayesian Information and Linkage Disequilibrium Iteratively Nested Keyway (BLINK) model and run in R® 3.6.1 using the package ‘BLINK’ [57]. BLINK has been demonstrated to have an enhanced statistical power and to be more efficient compared to previously developed models [57]. LOD threshold was set to 3 [58].

The two FEM models in BLINK were defined as following.

- FEM (1): y_i_= M_i1_b_1_ + M_i2_b_2_ + …+ M_ik_b_k_ + M_ij_d_j_ + e_i_.
- FEM (2): y_i_= M_i1_b_1_ + M_i2_b_2_ + …+ M_ij_b_j_ + e_i_.

Where y: being the vector phenotype; M_i1_, M_i2_, …, M_ik_: the genotypes of k pseudo QTNs that were initially empty and with effects b_1_, b_2_, …, b_k_, respectively; M_ij_: the j^th^ genetic marker of the i^th^ sample; and e_i_: the residual having a distribution with mean zero and a variance σ^2^_e_. LD heatmaps were generated using the package ‘LDheatmap’ in R® 3.6.1 [59]. Overlapping SNP markers between different traits were visualized using a Venn diagram that was established using the online software program accessible at http://jvenn.toulouse.inra.fr/app/example.html.

### Candidate gene discovery

A 40-kb genomic region harboring each significant SNP was used for searching candidate genes in the Phytozome 12 database (https://phytozome.jgi.doe.gov/), with an emphasis on those with functional annotations relevant to abiotic stresses.

### Epistatic interaction modelling

Pairwise epistatic interaction analysis (SNP * SNP interaction) was conducted using PLINK v1.07 [60]. The command line for conducting epistasis analysis in PLINK was ‘plink --file mydata --epistasis’. The interaction effect of two SNPs was estimated using the following model [60].

$$E\left[\mathrm Y\vert{\mathrm{Snp}}_{\mathrm i},\;{\mathrm{Snp}}_{\mathrm j}\right]\;=\;\beta_0\:+\:\beta_{\mathrm i}{\mathrm{Snp}}_{\mathrm i}\:+\:\beta_j{\mathrm{Snp}}_{\mathrm j}\:+\:\beta_{ij}\;\left({\mathrm{Snp}}_{\mathrm i}\;\mathrm X\;{\mathrm{Snp}}_{\mathrm j}\right)$$

with E[Y|Snp_i_, Snp_j_] being the vector of expected values for the response given the SNP data, β_0_ being the intercept, β_i_ being the main effect for the Snp_i_, β_j_ being the main effect for the Snp_j_, and β_ij_ being the interaction effect (epistasis) between Snp_i_ and Snp_j_. The parameter of interest in the above model was *β*_*ij*_ and the test to be conducted was H0: *β*_*ij*_ = 0. Choosing a minimum p-value for declaring a significant interaction effect can inflate the Type 1 error rate [61]. However, the current approach using various techniques for identifying a significant threshold while reducing the bias in estimating *β*_*ij*_ and limiting the Type 1 error rate could be still extremely computationally intensive. Therefore, we used an arbitrary threshold (p-value ≤ 10^− 6^) in this study given the great number of possible pairwise interactions and practical reasons during the data visualization process, while being biologically reasonable. Pairwise epistatic interaction was visualized using the package ‘circlize’ and run in R® 3.6.1 [56].

### Statement on Research involving plants

The study protocol complies with relevant institutional, national, and international guidelines and legislation.

The authors have permission to collect Cowpea [*Vigna unguiculata* (L.) Walp.].

## Supplementary Information

**Additional file 1: Table S1. **List of the MAGIC lines evaluated for salt tolerance along with their 8 founders (top 8 genotypes on the list), average number of dead plants per plot, the leaf injury score under salt treatment, SPAD chlorophyll under no-salt treatment, SPAD chlorophyll under salt treatment, and relative tolerance index (RTI) for SPAD chlorophyll. Sd represents the standard deviation across 4 replications. RTI was calculated as 100*(Phenotype_Stress/Phenotype_No_Stress). RTI was assessed for each replication and the RTI on the table was the average from each replication. **Table S2.** List of the MAGIC lines evaluated for salt tolerance along with their 8 founders (top 8 genotypes on the list), fresh leaf biomass under no-salt treatment, fresh leaf biomass under salt treatment, relative tolerance index (RTI) for fresh leaf biomass, fresh stem biomass under no-salt treatment, fresh stem under salt treatment, and relative tolerance index (RTI) for fresh stem biomass. Sd represents the standard deviation across 4 replications. Relative tolerance index (RTI) was calculated as 100*(Phenotype_Stress/Phenotype_No_Stress). RTI was assessed for each replication and the RTI on the table was the average from each replication. **Table S3.** List of the MAGIC lines evaluated for salt tolerance along with their 8 founders (top 8 genotypes on the list), total fresh above-ground biomass under no-salt treatment, total fresh above-ground biomass under salt treatment, relative tolerance index (RTI) for total fresh above-ground biomass, plant height under no-salt treatment, plant height under salt treatment, and relative tolerance index (RTI) for plant height. Sd represents the standard deviation across 4 replications. Relative tolerance index (RTI) was calculated as 100*(Phenotype_Stress/Phenotype_No_Stress). RTI was assessed for each replication and the RTI on the table was the average from each replication.

## Data Availability

The data that support the findings of this study are available in the supplementary material.
